# Supplementary figures and images for: Effective vaccine allocation strategies, balancing economy with infection control against COVID-19 in Japan
Source: PLoS One. 2021 Sep 2;16(9):e0257107. doi: 10.1371/journal.pone.0257107 (PMC8412346; doi:10.1371/journal.pone.0257107)

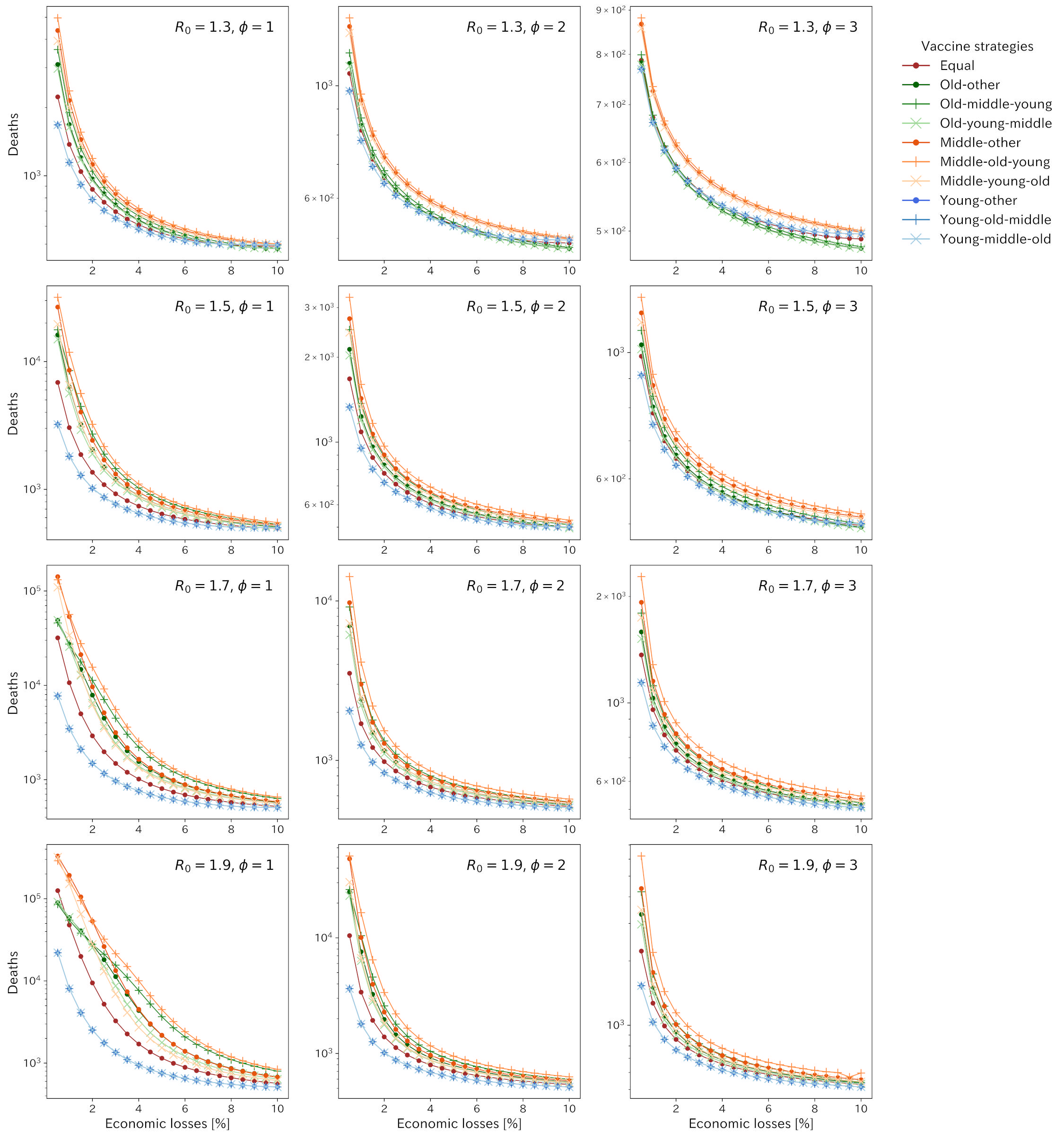

Supplement: S1 Fig — Each line shows vaccine allocation strategies. Three lines of young first vaccination strategies have overlain each other. (TIF) [file pone.0257107.s001.tif]

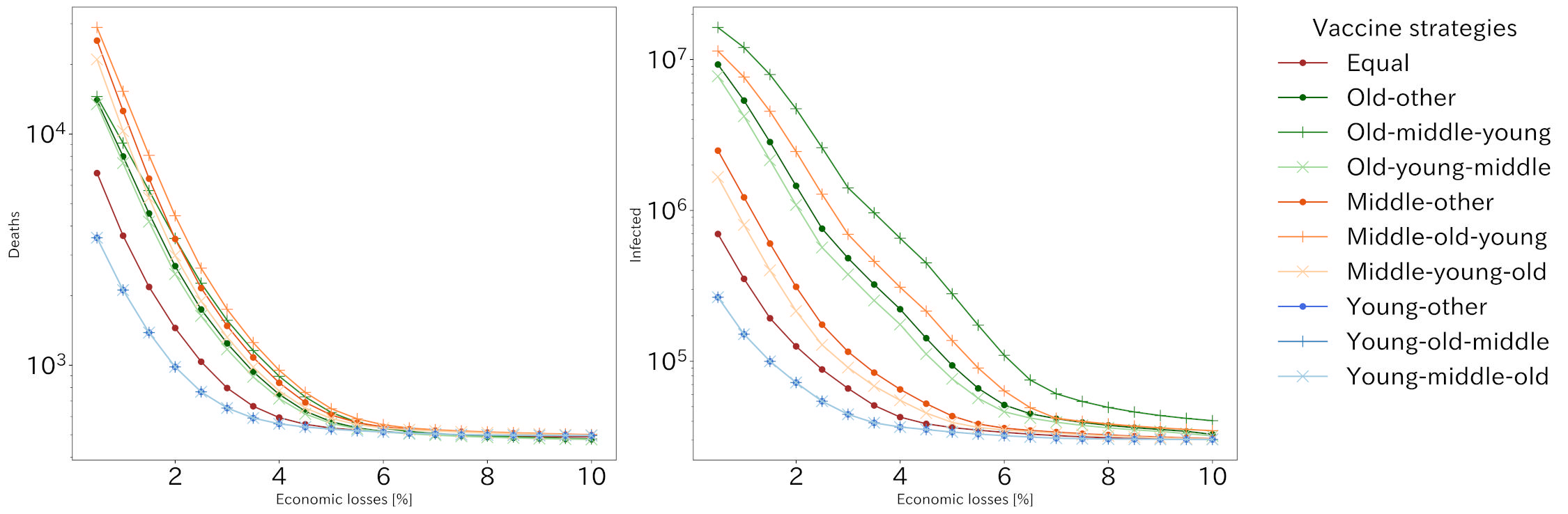

Supplement: S2 Fig — The optimized results of the cumulative number of deaths and infected population for each economic loss is presented. Three lines of young first vaccination strategies have overlain each other. The order of age groups on the label shows vaccine allocation strategies. (TIF) [file pone.0257107.s002.tif]

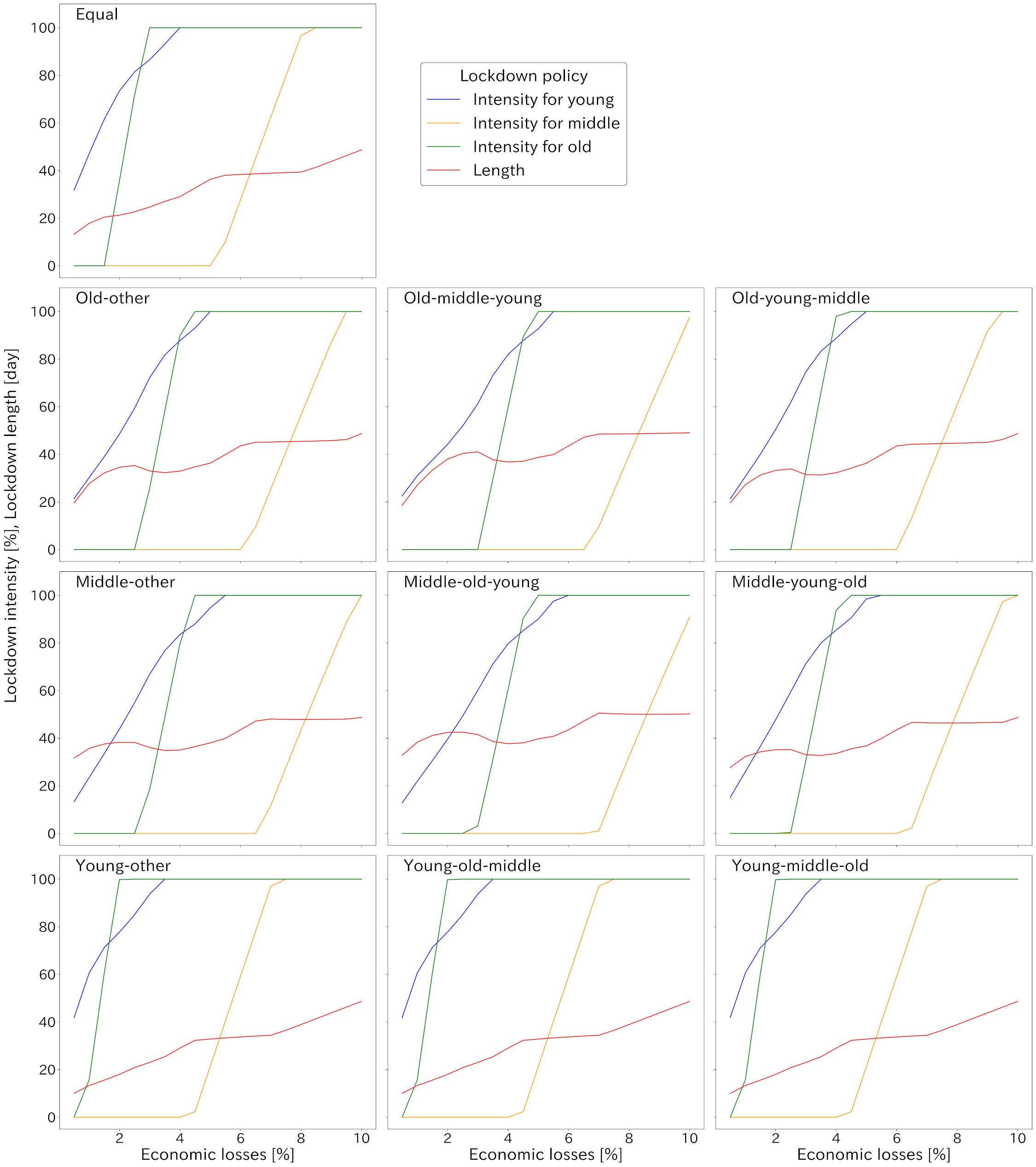

Supplement: S3 Fig — Each figure block contains lockdown intensities for young (blue), middle (yellow) and old (green) age group and lockdown length (red). It is noted that y axis represented lockdown intensity and lockdown length. (TIF) [file pone.0257107.s003.tif]

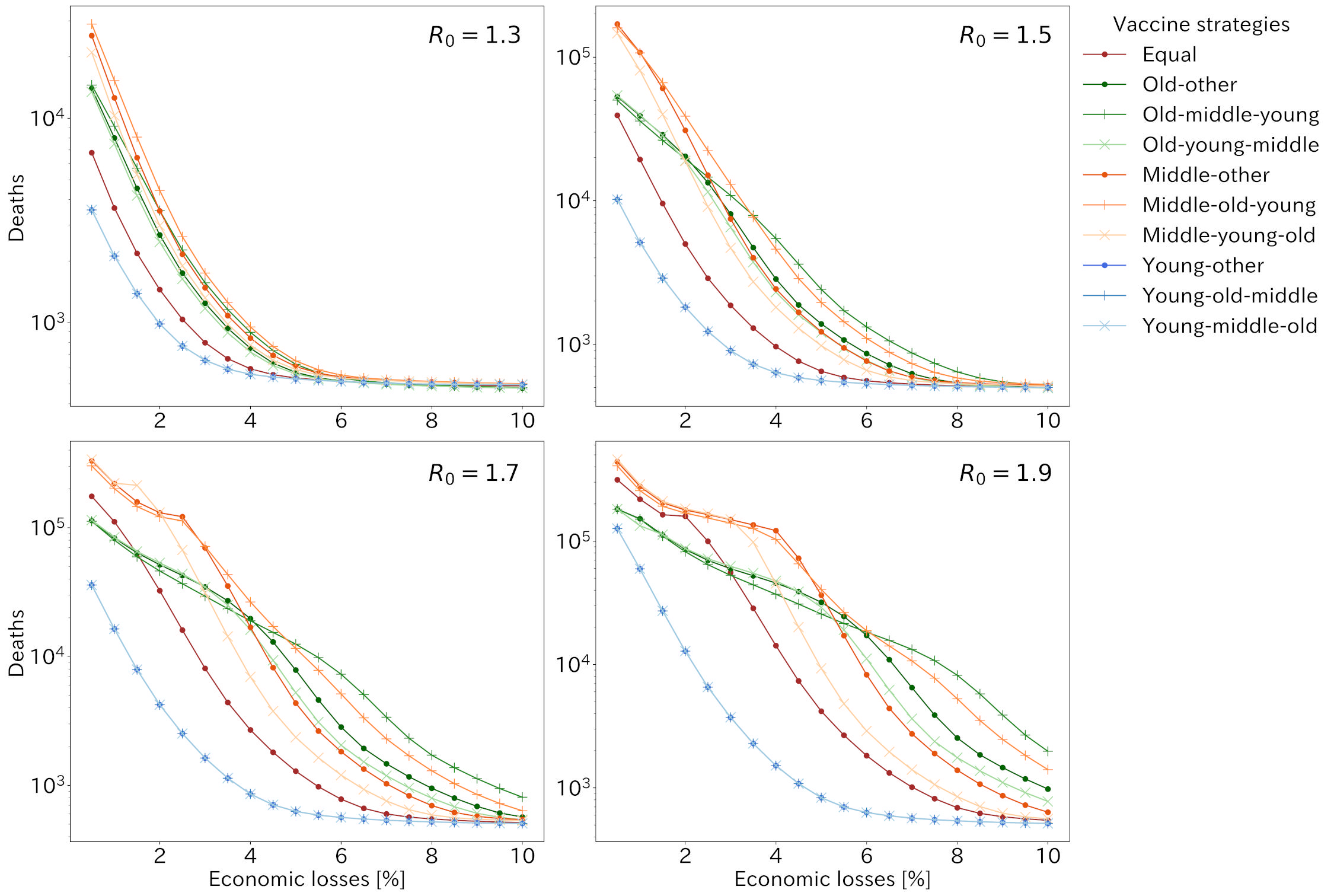

Supplement: S4 Fig — Each line shows vaccine allocation strategies. Three lines of young first vaccination strategies have overlain each other. (TIF) [file pone.0257107.s004.tif]

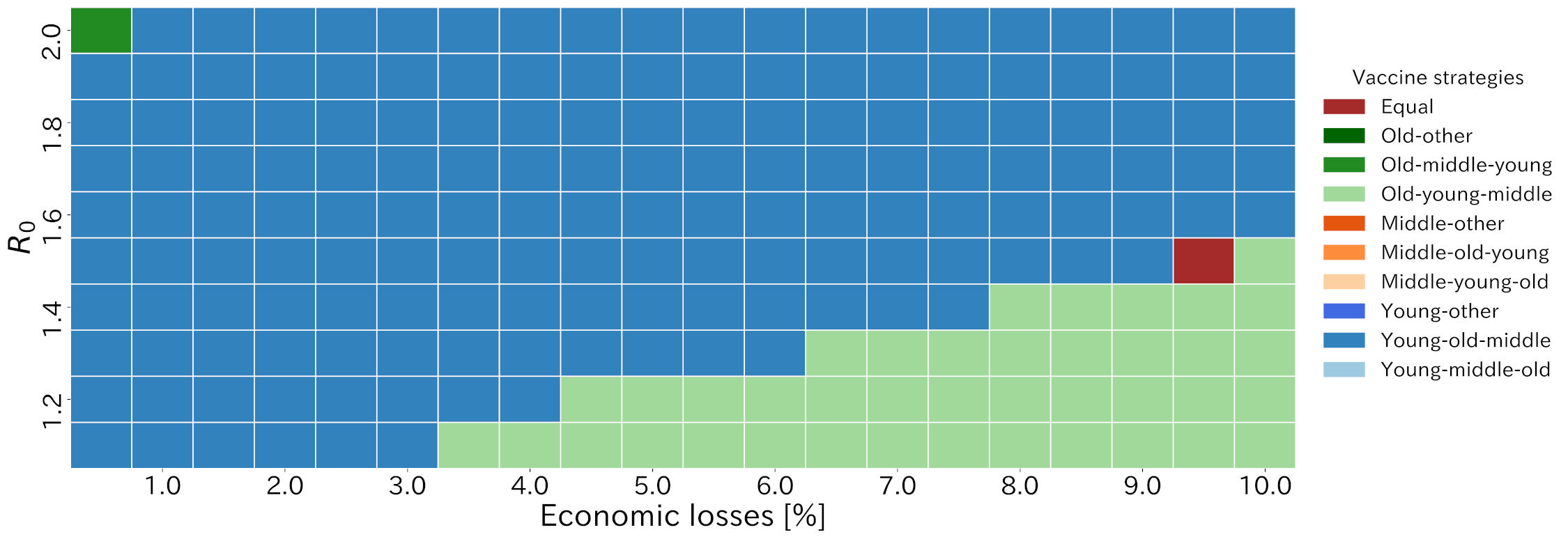

Supplement: S5 Fig — (TIF) [file pone.0257107.s005.tif]
