# Supplementary material for: Effective vaccine allocation strategies, balancing economy with infection control against COVID-19 in Japan
Source: PLoS One. 2021 Sep 2;16(9):e0257107. doi: 10.1371/journal.pone.0257107 (PMC8412346; doi:10.1371/journal.pone.0257107)
Supplement: S1 Table — (DOCX) [file pone.0257107.s006.docx]

**S1 Table.**

| ***Φ*** | ***R*_0_** | ***E* (*%*)** | **Vaccine strategy** | ***ts*** | ***T*_L_ (day)** | ***L* (%)** | **Infected** | **D** |
| --- | --- | --- | --- | --- | --- | --- | --- | --- |
| 0 | 1.1 | 1 | Young-old-middle | 0 | 9 | 48.82 | 103527 | 1360 |
| 0 | 1.1 | 4 | Young-old-middle | 0 | 19 | 100.00 | 35069 | 555 |
| 0 | 1.1 | 7 | Old-young-middle | 0 | 34 | 100.00 | 32596 | 477 |
| 0 | 1.1 | 10 | Old-young-middle | 0 | 48 | 100.00 | 30098 | 471 |
| 0 | 1.3 | 1 | Young-old-middle | 0 | 11 | 42.4 | 215683 | 2832 |
| 0 | 1.3 | 4 | Young-old-middle | 0 | 21 | 92.53 | 41935 | 646 |
| 0 | 1.3 | 7 | Young-old-middle | 0 | 34 | 100.00 | 30863 | 504 |
| 0 | 1.3 | 10 | Old-young-middle | 0 | 48 | 100.00 | 32003 | 477 |
| 0 | 1.5 | 1 | Young-old-middle | 0 | 12 | 37.59 | 578959 | 8033 |
| 0 | 1.5 | 4 | Young-old-middle | 0 | 23 | 82.94 | 59145 | 892 |
| 0 | 1.5 | 7 | Young-old-middle | 0 | 34 | 100.00 | 32014 | 520 |
| 0 | 1.5 | 10 | Equal | 0 | 48 | 100.00 | 31088 | 496 |
| 0 | 1.7 | 1 | Young-old-middle | 0 | 13 | 35.51 | 1871096 | 28194 |
| 0 | 1.7 | 4 | Young-old-middle | 0 | 26 | 73.62 | 108445 | 1659 |
| 0 | 1.7 | 7 | Young-old-middle | 0 | 34 | 100.00 | 34522 | 559 |
| 0 | 1.7 | 10 | Young-old-middle | 0 | 48 | 100.00 | 31079 | 506 |
| 0 | 1.9 | 1 | Young-old-middle | 0 | 13 | 35.46 | 6287080 | 102056 |
| 0 | 1.9 | 4 | Young-old-middle | 0 | 29 | 66.77 | 267658 | 4372 |
| 0 | 1.9 | 7 | Young-old-middle | 0 | 34 | 98.03 | 41180 | 671 |
| 0 | 1.9 | 10 | Young-old-middle | 0 | 48 | 100.00 | 31654 | 514 |
| 1 | 1.1 | 1 | Young-old-middle | 0 | 31 | 39.05 | 60150 | 839 |
| 1 | 1.1 | 4 | Old-young-middle | 0 | 30 | 79.48 | 45186 | 531 |
| 1 | 1.1 | 7 | Old-young-middle | 0 | 34 | 100.00 | 32596 | 477 |
| 1 | 1.1 | 10 | Old-young-middle | 0 | 48 | 100.00 | 30098 | 471 |
| 1 | 1.3 | 1 | Young-old-middle | 0 | 43 | 33.51 | 85457 | 1143 |
| 1 | 1.3 | 4 | Young-old-middle | 0 | 31 | 78.97 | 37312 | 581 |
| 1 | 1.3 | 7 | Young-old-middle | 0 | 34 | 100.00 | 30863 | 504 |
| 1 | 1.3 | 10 | Old-young-middle | 0 | 48 | 100.00 | 32003 | 477 |
| 1 | 1.5 | 1 | Young-old-middle | 0 | 56 | 29.37 | 138197 | 1809 |
| 1 | 1.5 | 4 | Young-old-middle | 0 | 38 | 71.04 | 42750 | 648 |
| 1 | 1.5 | 7 | Young-old-middle | 0 | 34 | 99.35 | 32032 | 520 |
| 1 | 1.5 | 10 | Equal | 0 | 48 | 100.00 | 31088 | 496 |
| 1 | 1.7 | 1 | Young-old-middle | 0 | 69 | 26.47 | 260491 | 3461 |
| 1 | 1.7 | 4 | Young-old-middle | 0 | 47 | 63.8 | 51573 | 758 |
| 1 | 1.7 | 7 | Young-old-middle | 0 | 39 | 92.51 | 34240 | 548 |
| 1 | 1.7 | 10 | Young-old-middle | 0 | 48 | 100 | 31079 | 506 |
| 1 | 1.9 | 1 | Young-old-middle | 0 | 81 | 24.45 | 574913 | 8053 |
| 1 | 1.9 | 4 | Young-old-middle | 0 | 58 | 57.81 | 66192 | 942 |
| 1 | 1.9 | 7 | Young-old-middle | 0 | 46 | 85.6 | 37600 | 591 |
| 1 | 1.9 | 10 | Young-old-middle | 0 | 48 | 100.00 | 31654 | 514 |

**S1 Table (Cont.).**

| ***Φ*** | ***R*_0_** | ***E* (*%*)** | **Vaccine strategy** | ***ts*** | ***T*_L_ (day)** | ***L* (%)** | **Infected** | **D** |
| --- | --- | --- | --- | --- | --- | --- | --- | --- |
| 2 | 1.1 | 1 | Old-young-middle | 0 | 46 | 47.19 | 62068 | 657 |
| 2 | 1.1 | 4 | Old-young-middle | 0 | 34 | 82.38 | 38116 | 509 |
| 2 | 1.1 | 7 | Old-young-middle | 0 | 34 | 100.00 | 32596 | 477 |
| 2 | 1.1 | 10 | Old-young-middle | 0 | 48 | 100.00 | 30098 | 471 |
| 2 | 1.3 | 1 | Young-old-middle | 0 | 45 | 47.59 | 55058 | 780 |
| 2 | 1.3 | 4 | Young-old-middle | 0 | 35 | 82.13 | 34807 | 550 |
| 2 | 1.3 | 7 | Old-young-middle | 0 | 45 | 90.53 | 37793 | 498 |
| 2 | 1.3 | 10 | Old-young-middle | 0 | 48 | 100.00 | 32003 | 477 |
| 2 | 1.5 | 1 | Young-old-middle | 0 | 55 | 44.32 | 69699 | 952 |
| 2 | 1.5 | 4 | Young-old-middle | 0 | 41 | 77.66 | 37526 | 582 |
| 2 | 1.5 | 7 | Young-old-middle | 0 | 38 | 96.41 | 31950 | 517 |
| 2 | 1.5 | 10 | Old-young-middle | 0 | 58 | 93.86 | 36666 | 495 |
| 2 | 1.7 | 1 | Young-old-middle | 0 | 67 | 41.66 | 94205 | 1247 |
| 2 | 1.7 | 4 | Young-old-middle | 0 | 48 | 73.52 | 41221 | 626 |
| 2 | 1.7 | 7 | Young-old-middle | 0 | 43 | 92.27 | 33424 | 535 |
| 2 | 1.7 | 10 | Young-old-middle | 0 | 48 | 100.00 | 31079 | 506 |
| 2 | 1.9 | 1 | Young-old-middle | 0 | 78 | 39.58 | 137931 | 1798 |
| 2 | 1.9 | 4 | Young-old-middle | 0 | 57 | 69.87 | 46233 | 685 |
| 2 | 1.9 | 7 | Young-old-middle | 0 | 49 | 88.35 | 35348 | 558 |
| 2 | 1.9 | 10 | Young-old-middle | 0 | 48 | 100.00 | 31654 | 514 |
| 3 | 1.1 | 1 | Old-young-middle | 0 | 45 | 57.22 | 47966 | 587 |
| 3 | 1.1 | 4 | Old-young-middle | 0 | 36 | 85.18 | 35397 | 499 |
| 3 | 1.1 | 7 | Old-young-middle | 0 | 35 | 98.98 | 32180 | 477 |
| 3 | 1.1 | 10 | Old-young-middle | 0 | 48 | 100.00 | 30098 | 471 |
| 3 | 1.3 | 1 | Old-young-middle | 0 | 70 | 51.33 | 62315 | 666 |
| 3 | 1.3 | 4 | Old-young-middle | 0 | 52 | 78.05 | 40450 | 526 |
| 3 | 1.3 | 7 | Old-young-middle | 0 | 47 | 91.88 | 35333 | 492 |
| 3 | 1.3 | 10 | Old-young-middle | 0 | 48 | 100.00 | 32003 | 477 |
| 3 | 1.5 | 1 | Young-old-middle | 0 | 53 | 54.81 | 52267 | 747 |
| 3 | 1.5 | 4 | Young-old-middle | 0 | 43 | 82.02 | 35407 | 556 |
| 3 | 1.5 | 7 | Old-young-middle | 0 | 64 | 85.2 | 38092 | 514 |
| 3 | 1.5 | 10 | Old-young-middle | 0 | 60 | 94.69 | 34735 | 491 |
| 3 | 1.7 | 1 | Young-old-middle | 0 | 63 | 52.61 | 62195 | 861 |
| 3 | 1.7 | 4 | Young-old-middle | 0 | 49 | 79.19 | 37697 | 583 |
| 3 | 1.7 | 7 | Young-old-middle | 0 | 45 | 93.1 | 32900 | 528 |
| 3 | 1.7 | 10 | Young-old-middle | 0 | 48 | 100.00 | 31079 | 506 |
| 3 | 1.9 | 1 | Young-old-middle | 0 | 73 | 50.76 | 76797 | 1033 |
| 3 | 1.9 | 4 | Young-old-middle | 0 | 56 | 76.64 | 40563 | 616 |
| 3 | 1.9 | 7 | Young-old-middle | 0 | 50 | 90.44 | 34282 | 544 |
| 3 | 1.9 | 10 | Young-old-middle | 0 | 48 | 100.00 | 31654 | 514 |
