# Supplementary material for: Effective vaccine allocation strategies, balancing economy with infection control against COVID-19 in Japan
Source: PLoS One. 2021 Sep 2;16(9):e0257107. doi: 10.1371/journal.pone.0257107 (PMC8412346; doi:10.1371/journal.pone.0257107)
Supplement: S2 Table — (DOCX) [file pone.0257107.s007.docx]

**S2 Table.**

| ***Φ*** | ***R*_0_** | ***E* (*%*)** | **Vaccine strategy** | ***ts*** | ***T*_L_ (day)** | ***L* (%)** | **Infected** | **Deaths** |
| --- | --- | --- | --- | --- | --- | --- | --- | --- |
| 0 | 1.1 | 1 | Old-young-middle | 0 | 11 | 41.70 | 493150 | 1803 |
| 0 | 1.1 | 4 | Old-young-middle | 0 | 20 | 93.02 | 67613 | 572 |
| 0 | 1.1 | 7 | Old-young-middle | 0 | 34 | 100.00 | 32596 | 477 |
| 0 | 1.1 | 10 | Old-young-middle | 0 | 48 | 100.00 | 30098 | 471 |
| 0 | 1.3 | 1 | Old-young-middle | 0 | 17 | 27.15 | 6625542 | 11320 |
| 0 | 1.3 | 4 | Old-young-middle | 0 | 29 | 65.69 | 487494 | 1237 |
| 0 | 1.3 | 7 | Old-young-middle | 0 | 34 | 100.00 | 63594 | 524 |
| 0 | 1.3 | 10 | Old-young-middle | 0 | 48 | 100.00 | 32003 | 477 |
| 0 | 1.5 | 1 | Old-middle-young | 0 | 19 | 24.46 | 36433603 | 45448 |
| 0 | 1.5 | 4 | Old-young-middle | 0 | 48 | 40.03 | 7013835 | 10403 |
| 0 | 1.5 | 7 | Old-young-middle | 0 | 48 | 70.71 | 435368 | 1113 |
| 0 | 1.5 | 10 | Old-young-middle | 0 | 48 | 100.00 | 55227 | 512 |
| 0 | 1.7 | 1 | Old-middle-young | 0 | 22 | 21.75 | 49643477 | 101793 |
| 0 | 1.7 | 4 | Old-middle-young | 0 | 46 | 41.48 | 41120259 | 35097 |
| 0 | 1.7 | 7 | Old-young-middle | 0 | 75 | 45.27 | 5668284 | 8912 |
| 0 | 1.7 | 10 | Old-young-middle | 0 | 63 | 76.57 | 298147 | 932 |
| 0 | 1.9 | 1 | Old-middle-young | 26 | 38 | 12.49 | 57968065 | 188281 |
| 0 | 1.9 | 4 | Old-middle-young | 0 | 47 | 40.59 | 52121061 | 61744 |
| 0 | 1.9 | 7 | Old-middle-young | 0 | 69 | 49.34 | 43285717 | 29872 |
| 0 | 1.9 | 10 | Old-young-middle | 0 | 93 | 51.86 | 3290884 | 5819 |
| 1 | 1.1 | 1 | Old-young-middle | 0 | 46 | 32.38 | 117486 | 869 |
| 1 | 1.1 | 4 | Old-young-middle | 0 | 30 | 79.48 | 45186 | 531 |
| 1 | 1.1 | 7 | Old-young-middle | 0 | 34 | 100.00 | 32596 | 477 |
| 1 | 1.1 | 10 | Old-young-middle | 0 | 48 | 100.00 | 30098 | 471 |
| 1 | 1.3 | 1 | Old-young-middle | 0 | 96 | 22.50 | 383751 | 1633 |
| 1 | 1.3 | 4 | Old-young-middle | 0 | 53 | 60.59 | 72983 | 631 |
| 1 | 1.3 | 7 | Old-young-middle | 0 | 42 | 89.60 | 44226 | 509 |
| 1 | 1.3 | 10 | Old-young-middle | 0 | 48 | 100.00 | 32003 | 477 |
| 1 | 1.5 | 1 | Old-young-middle | 0 | 148 | 18.12 | 2303807 | 5639 |
| 1 | 1.5 | 4 | Old-young-middle | 0 | 90 | 46.37 | 122606 | 855 |
| 1 | 1.5 | 7 | Old-young-middle | 0 | 65 | 71.92 | 58971 | 583 |
| 1 | 1.5 | 10 | Old-young-middle | 0 | 55 | 93.40 | 41625 | 503 |
| 1 | 1.7 | 1 | Old-young-middle | 0 | 166 | 17.08 | 14359555 | 25646 |
| 1 | 1.7 | 4 | Old-young-middle | 0 | 129 | 38.74 | 270321 | 1395 |
| 1 | 1.7 | 7 | Old-young-middle | 0 | 95 | 59.78 | 81625 | 711 |
| 1 | 1.7 | 10 | Old-young-middle | 0 | 76 | 79.61 | 50593 | 558 |
| 1 | 1.9 | 1 | Old-middle-young | 0 | 138 | 18.78 | 44363423 | 54776 |
| 1 | 1.9 | 4 | Old-young-middle | 0 | 161 | 34.70 | 1070771 | 3340 |
| 1 | 1.9 | 7 | Old-young-middle | 0 | 124 | 52.21 | 130357 | 943 |
| 1 | 1.9 | 10 | Old-young-middle | 0 | 100 | 69.69 | 64007 | 641 |

**S3 Table (Cont.).**

| ***Φ*** | ***R*_0_** | ***E* (*%*)** | **Vaccine strategy** | ***ts*** | ***T*_L_ (day)** | ***L* (%)** | **Infected** | **Deaths** |
| --- | --- | --- | --- | --- | --- | --- | --- | --- |
| 2 | 1.1 | 1 | Old-young-middle | 0 | 46 | 47.19 | 62068 | 657 |
| 2 | 1.1 | 4 | Old-young-middle | 0 | 34 | 82.38 | 38116 | 509 |
| 2 | 1.1 | 7 | Old-young-middle | 0 | 34 | 100.00 | 32596 | 477 |
| 2 | 1.1 | 10 | Old-young-middle | 0 | 48 | 100.00 | 30098 | 471 |
| 2 | 1.3 | 1 | Old-young-middle | 0 | 78 | 39.50 | 99597 | 828 |
| 2 | 1.3 | 4 | Old-young-middle | 0 | 52 | 71.85 | 47292 | 553 |
| 2 | 1.3 | 7 | Old-young-middle | 0 | 45 | 90.53 | 37793 | 498 |
| 2 | 1.3 | 10 | Old-young-middle | 0 | 48 | 100.00 | 32003 | 477 |
| 2 | 1.5 | 1 | Old-young-middle | 0 | 120 | 34.33 | 180584 | 1195 |
| 2 | 1.5 | 4 | Old-young-middle | 0 | 77 | 63.08 | 57267 | 621 |
| 2 | 1.5 | 7 | Old-young-middle | 0 | 64 | 80.73 | 42674 | 532 |
| 2 | 1.5 | 10 | Old-young-middle | 0 | 58 | 93.86 | 36666 | 495 |
| 2 | 1.7 | 1 | Old-young-middle | 0 | 155 | 31.49 | 515485 | 2213 |
| 2 | 1.7 | 4 | Old-young-middle | 0 | 103 | 57.24 | 71693 | 719 |
| 2 | 1.7 | 7 | Old-young-middle | 0 | 85 | 73.64 | 48288 | 577 |
| 2 | 1.7 | 10 | Old-young-middle | 0 | 75 | 86.26 | 39906 | 522 |
| 2 | 1.9 | 1 | Old-young-middle | 0 | 180 | 29.98 | 2547727 | 6323 |
| 2 | 1.9 | 4 | Old-young-middle | 0 | 128 | 53.34 | 97830 | 871 |
| 2 | 1.9 | 7 | Old-young-middle | 0 | 105 | 68.61 | 55992 | 636 |
| 2 | 1.9 | 10 | Old-young-middle | 0 | 92 | 80.62 | 43860 | 555 |
| 3 | 1.1 | 1 | Old-young-middle | 0 | 45 | 57.22 | 47966 | 587 |
| 3 | 1.1 | 4 | Old-young-middle | 0 | 36 | 85.18 | 35397 | 499 |
| 3 | 1.1 | 7 | Old-young-middle | 0 | 35 | 98.98 | 32180 | 477 |
| 3 | 1.1 | 10 | Old-young-middle | 0 | 48 | 100.00 | 30098 | 471 |
| 3 | 1.3 | 1 | Old-young-middle | 0 | 70 | 51.33 | 62315 | 666 |
| 3 | 1.3 | 4 | Old-young-middle | 0 | 52 | 78.05 | 40450 | 526 |
| 3 | 1.3 | 7 | Old-young-middle | 0 | 47 | 91.88 | 35333 | 492 |
| 3 | 1.3 | 10 | Old-young-middle | 0 | 48 | 100.00 | 32003 | 477 |
| 3 | 1.5 | 1 | Old-young-middle | 0 | 100 | 46.86 | 82018 | 792 |
| 3 | 1.5 | 4 | Old-young-middle | 0 | 73 | 71.86 | 45202 | 564 |
| 3 | 1.5 | 7 | Old-young-middle | 0 | 64 | 85.20 | 38092 | 514 |
| 3 | 1.5 | 10 | Old-young-middle | 0 | 60 | 94.69 | 34735 | 491 |
| 3 | 1.7 | 1 | Old-young-middle | 0 | 130 | 43.97 | 120121 | 1002 |
| 3 | 1.7 | 4 | Old-young-middle | 0 | 93 | 67.47 | 50936 | 612 |
| 3 | 1.7 | 7 | Old-young-middle | 0 | 82 | 80.25 | 41009 | 541 |
| 3 | 1.7 | 10 | Old-young-middle | 0 | 75 | 89.50 | 36674 | 509 |
| 3 | 1.9 | 1 | Old-young-middle | 0 | 156 | 41.97 | 220138 | 1417 |
| 3 | 1.9 | 4 | Old-young-middle | 0 | 113 | 64.34 | 58857 | 673 |
| 3 | 1.9 | 7 | Old-young-middle | 0 | 98 | 76.64 | 44657 | 573 |
| 3 | 1.9 | 10 | Old-young-middle | 0 | 90 | 85.61 | 38930 | 530 |
