# Supplementary material for: Effective vaccine allocation strategies, balancing economy with infection control against COVID-19 in Japan
Source: PLoS One. 2021 Sep 2;16(9):e0257107. doi: 10.1371/journal.pone.0257107 (PMC8412346; doi:10.1371/journal.pone.0257107)
Supplement: Attachment — Submitted filename: Reviewers report (PLOS).pdf [file pone.0257107.s009.pdf]

This manuscript analyzes using numerical simulation how the vaccine allocation strategies and the lockdown affect the economy of Japan. The authors showed on the social conditions of Japan that the economically optimum strategy changes depending on the basic reproduction number and acceptable economic loss. This paper is written in an academic style, but there are some technical problems to be solved before one considers suitability of the manuscript for publication.

1. From equations (1) and (2), the authors seem to implicitly assume that economic loss is proportional to the decrease of of effective reproduction number. This "linear assumption" seems crucial to the results of the paper, as the optimum condition is often around the complete lockdown "L=100%". In real social condition, the social cost may rapidly increase when the effective reproduction number, R, decreases. For example, compare the decrease of R "from 1.3 to 1.2" with "0.2 to 0.1". The increased economic (or social) loss may be much larger in the latter case. The discussion and formulation are found in the paper by Rowthorn and Maciejowski, (see Equation (12) of the link, https://www.ncbi.nlm.nih.gov/pmc/articles/PMC7499782/). The linear assumption adopted by the present authors is approximately the same as Rowthorn et al if the decrease of "R" is limited to be small. However, it is not the case of the present paper. The authors should consider how the "linear assumption" affects the results of the paper and discuss the validity of this assumption.

2. The explicit SEIR model with the effect of vaccination should be written in the form of a set of differential equations. SEIR model is a famous model in the field of theoretical epidemiology. However, the model the authors use is a modified one with the effect of vaccination. Please also note that PLOS ONE is a journal of multi-disciplinary. With the set of differential equations, one can check if the conservation of population is satisfied.

3. The reviewer could not find which curve correspond to which strategy in Figures 3 and 4. The author may use broken lines, dotted lines etc here. The author should find a way of drawing curves clearer.

4. The following are the minor points.
   Abbreviations must be defined when it first appears.

   (a) Line 100: R_0

   (b) Line 114: p_y and p_m even if they are defined in the caption of the figure

5. Line 103: python package, scipy should be Python package, Scipy

6. Line 134: "This kind of " should be replaced by more explicit words.

7. Line 145: Please explain why the authors use the age interval of five?

8. Line 154 Which paper corresponds to“reference paper”? Is it [25]?
9. Line 180: “The start timing of the lockdown, t_s, was usually 0 for almost all simulations.” “usually” should not be used here, as it is essential for the results. It must be written explicitly.
10. In figure 2: “1e4” is found in the figure. Is it 10^4? It should be written explicitly.
