# Supplementary material for: Effective vaccine allocation strategies, balancing economy with infection control against COVID-19 in Japan
Source: PLoS One. 2021 Sep 2;16(9):e0257107. doi: 10.1371/journal.pone.0257107 (PMC8412346; doi:10.1371/journal.pone.0257107)
Supplement: Attachment — Submitted filename: Reviewer Report - recommendation.docx [file pone.0257107.s008.docx]

**Peer review**

**Reviewer Report**

(Recommendation)

I recommend the research article. However, it would be appropriate for the authors to consider some recommendations.

**1. Summary of the investigation:**

The article uses the SEIR model. Apply an optimization method with the differential evolution method and use a proven Python algorithm. The research question: What is the most appropriate vaccination strategy based on economic cost? and of course, it assumes other variables or parameters that the literature and evidence review suggests. In this context, it seeks to balance the economy and infection control in Japan.

One of the greatest strengths is in having a proven methodology that is better than other existing ones. But as a weakness is that no appendix shows how the theoretical-mathematical model derives, and secondly, to be able to replicate the results in Python. Of course, this situation does not affect the results.

The main result of the study is that the effective vaccination strategy that prioritizes the younger generation outperformed the other strategies in terms of deaths, explained by the contact rate and therefore the transmission.

**2. Evidence and examples:**

First, a review of the literature on the current situation of the Vaccine against the Pandemic would be important. It could expand the description of the state of the art of public health policies that have been carried out and compare not only at the level of Japan but at least at the Asian or the Asia Pacific and/or Global level.

Second, review of the empirical evidence that can be presented (Results) and compared (Discussion) in the present study. It is important to compare the results with others in the region, at the level of Asia, Asia-Pacific, at the level of developed countries or the world.
