# Supplementary material for: Effective vaccine allocation strategies, balancing economy with infection control against COVID-19 in Japan
Source: PLoS One. 2021 Sep 2;16(9):e0257107. doi: 10.1371/journal.pone.0257107 (PMC8412346; doi:10.1371/journal.pone.0257107)
Supplement: Attachment — Submitted filename: review_R1_26July2021.docx [file pone.0257107.s010.docx]

**Revision Note**

We are most grateful to you and the reviewers for the comments on our manuscript.

**Major revisions**

According to reviewer 1 and 3, we performed additional analysis changing the relationships between economic loss and lockdown intensity in the equation (1) (line 104) in the revised manuscript. This change affected many figures and tables so that we summarized major revisions here at first.

• We additionally performed the nonlinear relationship between economic loss and lockdown intensity in the equation (1) in the revised manuscript.

• When we reflected the nonlinear relationship, original code setting could not yield enough precision of results, so that the entire results were re-calculated with more accurate precision settings.

• The latter part of Abstract section and the first and the second paragraph in the Discussion section were changed based on the results obtained by changing equation (1) and improving accuracy.

• Figures and Tables were changed as the following table.

| Previous manuscript | Revised manuscript | Change points |
| --- | --- | --- |
| Fig 1. Schematic of SEIR model. | Fig 1. Schematic of SEIR model. | To simplify the mathematical notations, *Np*_j_/*T*_vac_*N*_j,u_ was replaced with *V*_j,u._ |
| Fig 2. Illustration of relationship between economic loss and lockdown intensity and length. | Fig 2. Illustration of relationship between economic loss and lockdown intensity and length. | No change. |
| Fig 3. Optimized results and parameters for R_0_=1.3. | Fig 3. Optimized results and parameters for R_0_=1.3. | Original codes were reviewed, and optimized parameters became smoothed. |
| Fig 4. Cumulative number of deaths varying R0. | Fig 4. Cumulative number of deaths varying R0. | Results were updated but no major changes. |
|  | Fig 5. Heatmap among all. | Results were added to show the effect of nonlinearity. |
| Fig 5. Heatmap among the old. | Fig 6. Heatmap among the old. | Results were added to show the effect of nonlinearity. |
|  | S1 Fig. Cumulative number of deaths varying R0. | Results were added to show the effect of nonlinearity. |
| S3 Fig. Optimized results with different intensities of lockdown. | S2 Fig. Optimized results with different intensities of lockdown. | Results were updated but no major changes. |
| S4 Fig. Optimized parameters of different intensities of lockdown. | S3 Fig. Optimized parameters of different intensities of lockdown. | Results were updated and parameters became smoothed. |
|  | S4 Fig. Cumulative number of deaths varying R0 with different intensities of lockdown. | Added to compare with Fig 4. |
|  | S5 Fig. Heatmap among all with different intensities of lockdown. | Added to compare with Fig 5. |
| S1 Table. Numerical results among all. | S1 Table. Numerical results among all. | Results were added to show the effect of nonlinearity. |
| S2 Table. Numeric results among old | S2 Table. Numerical | Results were added to show the effect of nonlinearity. |

The rest of revised points were summarized in the context of point-by-point responses according to reviewer comments. Our responses were colored in red, and reviewers’ comments were colored in black.

**Response to PLOS ONE journal**

**Point 1.** Please ensure that your manuscript meets PLOS ONE's style requirements, including those for file naming. The PLOS ONE style templates can be found at

https://journals.plos.org/plosone/s/file?id=wjVg/PLOSOne_formatting_sample_main_body.pdf and

<https://journals.plos.org/plosone/s/file?id=ba62/PLOSOne_formatting_sample_title_authors_affiliations.pdf>

**Response 1:** According to guidelines, we changed our names of figures and tables for supplementary.

**Point 2:** 2. Thank you for stating the following financial disclosure:

"The funders had no role in study design, data collection and analysis, decision to publish, or preparation of the manuscript."

At this time, please address the following queries:

Please clarify the sources of funding (financial or material support) for your study. List the grants or organizations that supported your study, including funding received from your institution.

State what role the funders took in the study. If the funders had no role in your study, please state: “The funders had no role in study design, data collection and analysis, decision to publish, or preparation of the manuscript.”

If any authors received a salary from any of your funders, please state which authors and which funders.

If you did not receive any funding for this study, please state: “The authors received no specific funding for this work.”

Please include your amended statements within your cover letter; we will change the online submission form on your behalf.

**Response 2:** We included “The authors received no specific funding for this work.” in the cover letter. Thanks for changing the online submission form on our behalf.

**Response to Reviewer 1**

**Point 1:** The paper is on "Effective vaccine allocation strategies, balancing economy with infection control against COVID-19 in Japan". There were several articles published on that topic this year, and I do not think that this paper enters the top 20%. There is nothing really new here. Here are some comments

1)　(42) "COVID-19 has affected 42 tens of millions of people" it might depend on what "affect" means but if we include lockdowns for instance, it would be more "billions"

**Response 1:** Thank you for your suggestions and points to our study. We could improve our paper by your suggestions.
**Changes:** We changed the expressions in the Introduction section. 
“Over one hundred million people worldwide have been infected with COVID-19 since it was declared to be a pandemic by the World Health Organization on March 11th, 2020.” (line 43-45)

**Point 2:** 2)　(58) the grammar "vaccination in healthcare workers" is odd, but I am not a native English spearker

**Response 2:** We changed “in” into “for”.

**Changes:** We changed the expressions in the Introduction section.

“In Japan, vaccination for healthcare workers has begun in March 2021, and vaccination for the general elderly population has been implemented from April 12th, 2021. ” (line 65-66)

**Point 3:** 3) (88) "lockdown was performed only once" is that a relevent assumption ? so far, many countries have faced second or third wave, so it would make sense to assume that multiple lockdown is possible.

**Response 3:** Since the population was constantly vaccinated till the end of simulation, herd immunity was introduced by this vaccination. So that we considered that there was a small advantage in splitting the lockdown into separate parts and imposing the lockdown later. Then, we assumed that the lockdown would be imposed only once in our analysis. Since we did not explain this point, we have added it to the Method and to Discussion.

**Changes:** We added and changed explanations in the Simulation scenarios section.  
“Regarding lockdown strategies, it was assumed that the lockdown was performed only once during each simulation since there was a small chance of resurgence of COVID-19 at the latter phase of the simulation due to vaccination.” (line 95-97)

**Changes:** We added explanation the Discussion section.  
“As for a lockdown, its intensity in the present study was assumed to be precisely controlled by policy makers but in practice the actual extent of the contact reduction may not be obvious until a lockdown is in practice. Although the one-time lockdown assumption was adopted in this study, this implementation should be caution in the real-world settings. The simple SEIR model study [19] showed constant strong lockdown strategy was not optimal if the vaccination was started at the end of the simulation. The gradual relaxation strategy was preferred, and this strategy was conducted in the UK and Israel during vaccination. For the lockdown with age-specific intensities, the results were not much changed from the lockdown with the same intensity to all age groups (S5 Fig). The one exceptional result in S5 Fig when *R­*_0_ took 2.0 and economic loss was 0.5% indicated old-first strategy started to beat other strategies in case of higher *R*_0_ with weak lockdown.” (line 325-334)

**Point 4:** Furhermore I have major concern about the design of the lockdown : it is constant, over a given period of time, and uniform over the classes (same theta).

**Response 4:** As for your point of constant lockdown (same theta), we just use theta as lockdown efficacy, representing proportions of essential workers. In our model, intensity of lockdown, *L*_j_, was estimated. For the main result, it was constant whereas for the sensitivity analysis, lockdown was performed with different intensities for each generation. However, it was ambiguous what parameters were estimated in our paper. Then, we clarified it.
**Changes:** We added and changed explanations about fitting procedures in the Simulation scenarios section.
“We estimated parameters, *L*_j_ and *t*_s_, to minimize the cumulative number of deaths. *T*_L_ were calculated from *L*_j_ and *E* for each step. Ordinal differential equation was solved by Explicit Runge-Kutta method of order 5(4) [20] and optimization was done with differential evolution implemented in a Python package, Scipy version 1.5.3 [21,22].” (line 119-122)

Secondly, regarding the issue of the lockdown intensity being constant throughout the period, we assume that the lockdown measures are implemented based on the law. In fact, the content of emergency declarations in Japan is announced according to the law, and the countermeasures taken by the government during the period of emergency declarations did not change much over the period. As such, we assumed that lockdowns would be implemented with a constant intensity in this paper. In addition, this assumption is not completely in agreement with the actual situation, especially with regard to the stages of lockdown relaxation, as seen in Israel, the UK, and other countries where gradual relaxation has been implemented. This change was reflected in Response 3.

**Point 5:** Why ? In equation (1) the economic loss is linear... can't we assume that some people can work from home ? can't we assume that E is not linear in theta ? or N ? These assumptions are extremely strong, and not enough discuss. The problem is that all conclusions rely on those assumptions

**Response 5:** Regarding the issue of the validity of the linearity relationship between the lockdown intensity and economic impact, we agree with what you mentioned. In formulating our equation. we implicitly assumed that the reduction in human contact due to lockdown would have a direct economic impact on sales, however, as you pointed out, when the impact of teleworking and other factors is taken into account, it is not clear whether a linear relationship between the reduction in the amount of activity and the economic impact is right or not. Therefore, while the main results of the paper assumed linearity, we also performed additional calculations for the case where the economic loss, *E*, is a power function of the lockdown intensity as sensitivity analysis. The reason for adopting the power function form is based on the discussion in the reference [19] (Rowthorn R, Maciejowski J. A cost–benefit analysis of the COVID-19 disease. Oxford Review of Economic Policy. 2020;36: S38–S55. doi:10.1093/oxrep/graa030), in which the model assumes that mild countermeasures against infectious diseases such as teleworking can be taken without significant economic loss, but stronger measures such as the closure of companies in order to impose stronger behavioral restraint would result in a rapid increase in economic loss. However, it is not clear how appropriate it is to express the relationship between lockdown intensity and economic loss as a power function. The problem of this point is added in Discussion.

**Changes:** We added and changed explanations in the Simulation scenarios section.

“The relationship between lockdown intensities and economic loss was assumed to be linear ($\phi$=0) for the main results. Also, non-linear relationships expressed as a power function were applied. This non-linearity came from the discussion in [19]. In this setting, mild countermeasures against infectious diseases such as teleworking could be taken without significant economic loss, but stronger measures such as the closure of companies to achieve stronger behavioral restraint requirements would result in greater economic loss. Simulations with values of one, two or three for $\phi$ were also performed for the case of the lockdown imposed equally to each age group.” (line 108-115)

**Changes:** We added and changed the results and discussions as summarized in the table in the major revisions section in this revision note.

**Point 6:** 4) SEIR model (105) is that relevant for a COVID-19 type of pandemic ? what about asymptomatic ? that was the main challenge at first, and a strong motivation for lockdowns early 2020

**Response 6:** Since we focused on the number of deaths and asymptomatic infected cases can also be transmitted to others, asymptomatic cases are implicitly included in E and I compartment in this model. We have added a note on this point since it was not mentioned enough.

**Changes:** We added and changed explanations in the SEIR model scheme section.

“The third reason is that it is difficult for asymptomatic cases to be separated from susceptible ones without tests.” (line 147-148)

**Point 7:** 5) (128) again, things are uniform over classes... aren't there are more interactions within age classes ? Here having a similar theta is weird.

**Response 7:** As answered in Response 3, The difference in the frequency of interaction between generations is reflected in the contact matrix ρ.

**Response to Reviewer 2**

**Point 1:** I recommend the research article. However, it would be appropriate for the authors to consider some recommendations.

**1. Summary of the investigation:**

The article uses the SEIR model. Apply an optimization method with the differential evolution method and use a proven Python algorithm. The research question: What is the most appropriate vaccination strategy based on economic cost? and of course, it assumes other variables or parameters that the literature and evidence review suggests. In this context, it seeks to balance the economy and infection control in Japan.

One of the greatest strengths is in having a proven methodology that is better than other existing ones. But as a weakness is that no appendix shows how the theoretical-mathematical model derives, and secondly, to be able to replicate the results in Python. Of course, this situation does not affect the results.

The main result of the study is that the effective vaccination strategy that prioritizes the younger generation outperformed the other strategies in terms of deaths, explained by the contact rate and therefore the transmission.

**Response 1:** We are sorry for the use of the doctrine. Derivation of our model was explicitly written in the revised version. Also, we added method names for solving differential equations and optimization.

**Changes:** Differential equations have been added as shown in equation (2)-(11) in SEIR model scheme section.

“Equations of our SEIR model in the present study are shown as

$$\begin{aligned} \frac{dS_{j,u}\left( t \right)}{dt}=-\lambda_{j}\left( t \right)S_{j,u}\left( t \right)-V_{j,u}\left( t \right)S_{j,u}\left( t \right), \#\left( 2 \right) \\ \frac{dE_{j,u}\left( t \right)}{dt}=\lambda_{j}\left( t \right)S_{j,u}\left( t \right)-\tau E_{j,u}\left( t \right)-V_{j,u}\left( t \right)E_{j,u}\left( t \right), \#\left( 3 \right) \\ \frac{dI_{j,u}\left( t \right)}{dt}= \tau E_{j,u}\left( t \right)-\gamma I_{j,u}\left( t \right)-V_{j,u}\left( t \right)I_{j,u}\left( t \right), \#\left( 4 \right) \\ \frac{dR_{j,u}\left( t \right)}{dt}=\gamma\left( 1-{IFR}_{j} \right)I_{j,u}\left( t \right)-V_{j,u}\left( t \right)R_{j,u}\left( t \right), \#\left( 5 \right) \\ \frac{dD_{j,u}\left( t \right)}{dt}=\gamma{IFR}_{j}I_{j,u}\left( t \right)-V_{j,u}\left( t \right)D_{j,u}\left( t \right), \#\left( 6 \right) \\ \frac{dS_{j,v}\left( t \right)}{dt}=-(1-VE_{\lambda})\lambda_{j}\left( t \right)S_{j,v}\left( t \right)+V_{j,u}\left( t \right)S_{j,u}\left( t \right), \#\left( 7 \right) \\ \frac{dE_{j,v}\left( t \right)}{dt}=(1-VE_{\lambda})\lambda_{j}\left( t \right)S_{j,v}\left( t \right)-\tau E_{j,v}\left( t \right)+V_{j,u}\left( t \right)E_{j,u}\left( t \right), \#\left( 8 \right) \\ \frac{dI_{j,v}\left( t \right)}{dt}= \tau E_{j,v}\left( t \right)-\gamma I_{j,v}\left( t \right)+V_{j,u}\left( t \right)I_{j,u}\left( t \right), \#\left( 9 \right) \\ \frac{dR_{j,v}\left( t \right)}{dt}=\gamma\left( 1-\left( 1-VE_{d} \right){IFR}_{j} \right)I_{j,v}\left( t \right)+V_{j,u}\left( t \right)R_{j,u}\left( t \right), \#\left( 10 \right) \\ \frac{dD_{j,v}\left( t \right)}{dt}=\gamma\left( 1-VE_{d} \right){IFR}_{j}I_{j,v}\left( t \right)+V_{j,u}\left( t \right)D_{j,u}\left( t \right),\#(11) \end{aligned}$$

where

$$\begin{aligned} V_{j,u}\left( t \right)=\frac{Np_{j}\left( t \right)}{T_{vac}}\frac{1}{N_{j,u}\left( t \right)}, \#\left( 12 \right) \end{aligned}$$

and semantics of them are depicted in Fig 1.” (line 124-128)

**Changes:** We added and changed explanations about fitting procedures in the Simulation scenarios section.
“We estimated parameters, *L*_j_ and *t*_s_, to minimize the cumulative number of deaths. *T*_L_ were calculated from *L*_j_ and *E* for each step. Ordinal differential equation was solved by Explicit Runge-Kutta method of order 5(4) [20] and optimization was done with differential evolution implemented in a Python package, Scipy version 1.5.3 [21,22].” (line 119-122)

**Point 2: 2. Evidence and examples:**

First, a review of the literature on the current situation of the Vaccine against the Pandemic would be important. It could expand the description of the state of the art of public health policies that have been carried out and compare not only at the level of Japan but at least at the Asian or the Asia Pacific and/or Global level.

**Response 2:** For the first request, we added the current situation of major countries in the introduction.

**Changes:** We added in the introduction section.

“As of the 15th of June, 20.8% of the world's population has been vaccinated at least once, especially in many developed countries, where more than 40% of the population has already been vaccinated. However, in low-income countries, only 0.8% of the population has been vaccinated at least once, and vaccination in low-income countries is still an issue for the future. And as a consequence, some developed countries like Israel and UK have confirmed a decrease in the number of newly reported case of infection [3].”  (line 59-65)

**Point 3:** Second, review of the empirical evidence that can be presented (Results) and compared (Discussion) in the present study. It is important to compare the results with others in the region, at the level of Asia, Asia-Pacific, at the level of developed countries or the world.

**Response 3:** Since this study was calculated based on Japan's population structure by age, similar results may not be directly applicable to other countries. We added a comment to the discussion. (And also the way of implementation of lockdown.)

**Changes:** We added limitations in the Discussion section.

“Finally, since the calculations in this study are based on the population structure and contact rates in Japan, the results obtained are not directly applicable to foreign cases.”  (line 344-346)

**Response to Reviewer 3**

**Point 1:** This manuscript analyzes using numerical simulation how the vaccine allocation

strategies and the lockdown affect the economy of Japan. The authors

showed on the social conditions of Japan that the economically optimum

strategy changes depending on the basic reproduction number and acceptable

economic loss. This paper is written in an academic style, but there are some

technical problems to be solved before one considers suitability of the manuscript

for publication.

1. From equations (1) and (2), the authors seem to implicitly assume that economic loss is proportional to the decrease of of effective reproduction number. This “linear assumption” seems crucial to the results of the paper, as the optimum condition is often around the complete lockdown ”L=100%”. In real social condition, the social cost may rapidly increase when the effective reproduction number, R, decreases. For example, compare the decrease of R “from 1.3 to 1.2” with “0.2 to 0.1”. The increased economic (or social) loss may be much larger in the latter case. The discussion and formulation are found in the paper by Rowthorn and Maciejowski,(see Equation (12) of the link, https://www.ncbi.nlm.nih.gov/pmc/articles/PMC7499782/).The linear assumption adopted by the present authors is approximately the same as Rowthorn et al if the decrease of ”R” is limited to be small. However, it is not the case of the present paper. The authors should consider how the ”linear assumption” affects the results of the paper and discuss the validity of this assumption.

**Response 1:** Regarding the issue of the validity of the linearity relationship between the lockdown intensity and economic impact, we agree with what you mentioned. In formulating our equation. we implicitly assumed that the reduction in human contact due to lockdown would have a direct economic impact on sales, however, as you pointed out, when the impact of teleworking and other factors are taken into account, it is not clear whether a linear relationship between the reduction in the amount of activity and the economic impact is right or not. Therefore, while the main results of the paper are based on the assumption of linearity, we also performed additional calculations for the case where the economic loss, *E*, is a power function of the lockdown intensity as the paper you referred to for the sensitivity analysis. However, it is not clear how appropriate it is to express the relationship between lockdown intensity and economic loss as a power function. The problem of this point is added in Limitation.

**Changes:** We added and changed explanations in the Simulation scenarios section.

“The relationship between lockdown intensities and economic loss was assumed to be linear ($\phi$=0) for the main results. Also, non-linear relationships expressed as a power function were applied. This non-linearity came from the discussion in [19]. In this setting, mild countermeasures against infectious diseases such as teleworking could be taken without significant economic loss, but stronger measures such as the closure of companies to achieve stronger behavioral restraint requirements would result in greater economic loss. Simulations with values of one, two or three for $\phi$ were also performed for the case of the lockdown imposed equally to each age group.” (line 108-115)

**Changes:** We added and changed the results and discussions as summarized in the table in the major revisions section in this revision note.

**Point 2:** 2. The explicit SEIR model with the effect of vaccination should be written in the form of a set of differential equations. SEIR model is a famous model in the field of theoretical epidemiology. However, the model the authors use is a modified one with the effect of vaccination. Please also note that PLOS ONE is a journal of multi-disciplinary. With the set of differential equations, one can check if the conservation of population is satisfied.

**Response 2:** Revised as you requested.

**Changes:** Differential equations have been added as shown in equation (2)-(11) in SEIR model scheme section.
“Equations of our SEIR model in the present study are shown as

$$\begin{aligned} \frac{dS_{j,u}\left( t \right)}{dt}=-\lambda_{j}\left( t \right)S_{j,u}\left( t \right)-V_{j,u}\left( t \right)S_{j,u}\left( t \right), \#\left( 2 \right) \\ \frac{dE_{j,u}\left( t \right)}{dt}=\lambda_{j}\left( t \right)S_{j,u}\left( t \right)-\tau E_{j,u}\left( t \right)-V_{j,u}\left( t \right)E_{j,u}\left( t \right), \#\left( 3 \right) \\ \frac{dI_{j,u}\left( t \right)}{dt}= \tau E_{j,u}\left( t \right)-\gamma I_{j,u}\left( t \right)-V_{j,u}\left( t \right)I_{j,u}\left( t \right), \#\left( 4 \right) \\ \frac{dR_{j,u}\left( t \right)}{dt}=\gamma\left( 1-{IFR}_{j} \right)I_{j,u}\left( t \right)-V_{j,u}\left( t \right)R_{j,u}\left( t \right), \#\left( 5 \right) \\ \frac{dD_{j,u}\left( t \right)}{dt}=\gamma{IFR}_{j}I_{j,u}\left( t \right)-V_{j,u}\left( t \right)D_{j,u}\left( t \right), \#\left( 6 \right) \\ \frac{dS_{j,v}\left( t \right)}{dt}=-(1-VE_{\lambda})\lambda_{j}\left( t \right)S_{j,v}\left( t \right)+V_{j,u}\left( t \right)S_{j,u}\left( t \right), \#\left( 7 \right) \\ \frac{dE_{j,v}\left( t \right)}{dt}=(1-VE_{\lambda})\lambda_{j}\left( t \right)S_{j,v}\left( t \right)-\tau E_{j,v}\left( t \right)+V_{j,u}\left( t \right)E_{j,u}\left( t \right), \#\left( 8 \right) \\ \frac{dI_{j,v}\left( t \right)}{dt}= \tau E_{j,v}\left( t \right)-\gamma I_{j,v}\left( t \right)+V_{j,u}\left( t \right)I_{j,u}\left( t \right), \#\left( 9 \right) \\ \frac{dR_{j,v}\left( t \right)}{dt}=\gamma\left( 1-\left( 1-VE_{d} \right){IFR}_{j} \right)I_{j,v}\left( t \right)+V_{j,u}\left( t \right)R_{j,u}\left( t \right), \#\left( 10 \right) \\ \frac{dD_{j,v}\left( t \right)}{dt}=\gamma\left( 1-VE_{d} \right){IFR}_{j}I_{j,v}\left( t \right)+V_{j,u}\left( t \right)D_{j,u}\left( t \right),\#(11) \end{aligned}$$

where

$$\begin{aligned} V_{j,u}\left( t \right)=\frac{Np_{j}\left( t \right)}{T_{vac}}\frac{1}{N_{j,u}\left( t \right)}, \#\left( 12 \right) \end{aligned}$$

and semantics of them are depicted in Fig 1.” (line 124-128)

**Point 3:** 3. The reviewer could not find which curve correspond to which strategy in

Figures 3 and 4. The author may use broken lines, dotted lines etc here.

The author should find a way of drawing curves clearer.

**Response 3:** We added markers to each line and these lines can be distinguished. We attached Figure 3 here for example.

**Point 4:** 4. The following are the minor points.

Abbreviations must be defined when it first appears.

(a) Line 100: R0

(b) Line 114: py and pm even if they are defined in the caption of the

Figure

**Response 4:** Revised as you requested.

**Changes:** definitions of R0,pj,po,py,pm have been added in SEIR model scheme.

“basic reproduction number *R*_0_”(line 116)

“Vaccine allocation percentage for age group *j*, *p*_j_ ”(line 138)

“a mean vaccine allocation percentage for old generation, *p*_o_*_,_*” (line 140)

“mean vaccine allocation percentages for young, *p*_y_, and middle generations, *p*_m_,”　(line 141-142)

**Point 5:** 5. Line 103: python package, scipy should be Python package, Scipy

**Response 6:** Revised as requested.

**Point 6:** 6. Line 134: “This kind of ” should be replaced by more explicit words.

**Response 6:** Revised as requested.

**Changes:** We changed explanations in the Force of infection and basic reproduction number section.

“This derivation method of *R*_0_ was described in [24].” (line159-160)

**Point 7:** 7. Line 145: Please explain why the authors use the age interval of five?
**Response 7:** We added an explanation about the interval of five.
**Changes:** We added an explanation in the Parameter specification section.
“To truncate the original data to the values used in the model,”(line 170)

**Point 8:** 8. Line 154 Which paper corresponds to“reference paper”? Is it [25]?

**Response 8:** Revised as requested.

**Changes:** We added a reference number in the Parameter specification section.

“The contact rates of the reference paper [27] ”(line178-179)

**Point 9:** 9. Line 180: “The start timing of the lockdown, ts, was usually 0 for almost

all simulations.” “usually” should not be used here, as it is essential for

the results. It must be written explicitly.

**Response 9:** We clearly stated in which case ts became zero and not.

**Changes:** We added additional sentences about ts in the Result section.

“The start timing of the lockdown, *t*_s_, was estimated to be 0^th^ day for almost all simulations. *t*_s_ was estimated to be more than 0^th^ day (sometimes around 70^th^ day) when $\phi$ took a value of 0, *R*_0_ was higher, economic losses were lower, and the strategies were the middle first ones, the old first ones, or the equal strategy. In the case of a value of $\phi$ more than zero, *t*_s_ was estimated to be 0^th^ day for all cases.” (line 203-207)

**Changes:** We deleted the sentences about *t*_s_ since the above changes were introduced in the Result section.

“The optimized values of the start timing of the lockdown, *t*_s_, were the start timing of the simulation, that is, 0 for the most of simulations.”

**Point 10:** 10. In figure 2: “1e4” is found in the figure. Is it 10^4^? It should be written

explicitly.

**Response 10:** We changed “1e4” to 10^4^ in the z axis in Figure 2.
